# Analysis of *NSP4* Gene and Its Association with Genotyping of Rotavirus Group A in Stool Samples

**DOI:** 10.22034/ibj.22.1.42

**Published:** 2018-01

**Authors:** Ali Teimoori, Mehrab Nejati, Saeedeh Ebrahimi, Manoochehr Makvandi, Milad Zandi, Azarakhsh Azaran

**Affiliations:** Health Research Institute, Infectious and Tropical Diseases Research Center, Ahvaz Jundishapur University of Medical Sciences, Ahvaz, Iran

**Keywords:** *NSP4*, Rotavirus, Genotyping

## Abstract

**Background:**

Non-structural protein 4 (*NSP4*) is a critical protein for rotavirus (RV) replication and assembly. This protein has multiple domains and motifs that predispose its function and activity. *NSP4* has a sequence divergence in human and animal RVs. Recently, 14 genotypes (E1-E14) of *NSP4* have been identified, and E1 and E2 have been shown to be the most common genotypes in human.

**Methods:**

The gene and protein sequence of *NSP4* in RV-positive samples were inspected with the aim of *NSP4* genotyping and variation analysis in viroporin and other domains. P and G typings of RV samples were carried out by WHO primers using a semi-multiplex PCR method. Non-typeable RV samples were amplified by conserved primers and sequenced.

**Results:**

In viroporin and enterotoxin, conserved sequence was detected, and amino acids substitution with the same biochemical properties was found.

**Conclusion:**

Association of *NSP4* genotype with P or G genotyping G1/G9 correlates with E1 genogroups. In electrophoretyping of RV, E2 genotype had a short pattern when compared to E1.

## INTRODUCTION

Rotavirus (RV) is a major pathogen causing severe viral diarrhea in young children and animals and is associated with sporadic outbreaks of diarrhea in elderly and immune-compromised patients[1-3]. RV is a genus of the *Reoviridae* family with 100 nm diameter and contains icosahedral nucleocapsid symmetry[4]. Mature viral particles are composed of triple-layered protein capsids that enclose dsRNA genome with 11 segments. This genome encodes six structural proteins and seven NSPs, and the role of these proteins in the pathogenesis of RV is more or less accepted[4,5].

Segment 10 of RV encodes a non-structural protein (NSP), NSP4, which is a multifunctional and complex protein[6]. On the other hand, NSP4 is the first described viral enterotoxin that can induce “dose-dependent” and “age-dependent” diarrhea without histological changes in mice[7]. Increasing evidence shows that this enterotoxin activates signal transduction pathway and increases intracellular calcium levels in cells by mobilizing calcium from endoplasmic reticulum that leads to chloride secretion[8]. Pathophysiological mechanism of diarrhea includes destruction of RV-infected enterocytes, malabsorption, and neurotoxic activity induced by NSP4 enterotoxin[9]. This protein is important for RV morphogenesis and pathogenesis and efficiently stimulates immune response and can be targeted for the development of an effective vaccine[5,10].

RV is genetically divided into 14 genogroups from E1-E14. NSP4 is a relatively small protein with 175 amino acids (aa)[11]. NSP4 has been indicated to be an ER-specific transmembrane glycoprotein and acts as an intracellular receptor that binds to double-layered particles via C-terminus sequence, thereby facilitating ER entrance and acquisition of outer coating to form triple-layered particles[12-14]. An active synthetic peptide, 114-135 residues of NSP4, induces age-dependent diarrhea in mice; however, anti-NSP4-specific antibodies reduce the symptoms of diarrhea by neutralizing the enterotoxin effects[7]. Other functional and structural domains in NSP4 sequence include a coiled-coil domain and two viral protein-binding domains[15]. NSP4 contains two N-glycosylation sites in the amino terminus that are important to functionality.

The main aim of this study was to determine the genotypic diversity among RV-positive specimens based on sequencing and phylogenetic analysis of the gene coding for *NSP4*. Overlapping domains in the amino acid sequence were also determined and explained. Moreover, we determined the gene constellation of *NSP4* with P and G genotypes in our samples and also compared electrophoretypes with G or P genotyping.

## MATERIALS AND METHODS

### Samples

A total of 27 RV-positive samples were obtained from Virology Laboratory of Ahvaz Medical Science University (Ahvaz, Iran) from 2013 to 2015. Fecal samples were collected from children under five years old with diarrhea from Aboozar Children Hospital, Ahvaz. RV was detected in the samples using ELISA (Abbott Laboratories, Chicago, USA) and RT-PCR. The fecal samples were diluted to about 10% (wt/vol) in DMEM medium, and then an equal volume of trichlorotrifluoroethane (Freon) was added and clarified by a low speed centrifugation (~ 8000 ×g).

### RT-PCR amplification and sequencing of the NSP4 Gene

RV dsRNA was extracted using guanidine/ phenol solution according to the manufacturer’s instructions (Sinaclon, Iran). Thereafter, RNA pellets were dissolved in 50 µl of diethyl pyrocarbonate-treated water and stored at -70 °C until use. *NSP4* gene from group A RV was amplified by OneStep RT-PCR Kit (QIAGEN, Germany) with *NSP4* F-6 5’-TTAAAAGTTCTGTTCCGAGAGAGCG-3’ and *NSP4* R-6 5’-GTCACAYTAAGACCRTTCCTTCC AT-3’ primers.

### G and P genotyping

Genotyping of samples was performed according to the WHO Manual of RV detection and characterization methods with minimal modification at genotyping protocols. Semi-multiplex PCR was employed for the amplification of VP7 and VP4 genes. aBT1 G1, aCT2 G2, G3-Aust, aDT4 G4, aAT8 G8, and G9, as forward primers (Asian type), and End9, as a reverse primer, for VP7 amplification were used for genotyping by semi-multiplex PCR. VP4 gene amplification of different genotypes were performed by con3, as well as 2T-1 P[4], 1T-1 P[8], and 3T-1 P[6] forward and reverse primers, respectively. The cDNA was constructed with RevertAid First Strand cDNA Synthesis Kit (Life Technologies, USA) and gene specific consensus End 9 and con3 primers (1 µM final concentration). Typically, the first-round PCR was performed in a 50-µl volume containing 1 × PCR buffer (75 mM Tris/HCl [pH 8.8], 2 mM MgCl_2_, 200 µM dNTP mix, the appropriate primer mixture (500 nm each), 1 U Taq polymerase, and 5 µl cDNA. PCR reaction was run for 35 cycles of 94 °C for 45 s, 50 °C for 30 s, and 72 °C for 1 min, with a final extension step of 72 °C for 10 min.

### RNA electrophoretyping

SDS-PAGE was performed with slab gels using Laemmli’s method[16]. After electrophoresis, gels were fixed and stained simultaneously in a solution containing 5% ethanol, 1% nitric acid, and 0.1% AgNO_3_ for 5 min; thereafter, the solution was discarded. The gels were rinsed three times with distilled water for 10 s and then developed with a solution of 1.3% NaOH, 0.5% Na_2_CO_3_, and 0.4% HCOH (30%) for 1 to 2 min until the appearance of dark-stained bands on the yellow background[17]. Development was stopped with a solution containing 5% ethanol and 1% nitric acid for 1 min, and the stopping solution was then discarded[18,19].

## RESULTS

### NSP4 coding sequence analysis

Totally, 26 high-quality sequences were obtained from 27 samples of *NSP4* coding sequence gene. The sequences were submitted to the GenBank with accession numbers KT148598-KT148623. Based on the RV Classification Working Group, *NSP4* has 14 genotypes[20,21]. RotaC2.0 was utilized, as a regularly updated online automatic web application, for RV *NSP4* genotyping. The detected genotypes included E1 (20 cases, ~77%) and E2 (6 cases, ~23%). NetNGlyc 1.0 Server was employed for the prediction of N-glycosylation sites. Position 8 of *NSP4* protein sequence revealed N-glycosylation site patterns Asn, Tyr, Thr in E1 and E2 genotype but at position 18 in E1 and E2 genotype indicated patterns Asn, Asp, Thr and Asn, Ser, Thr, respectively.

**Fig.1:**
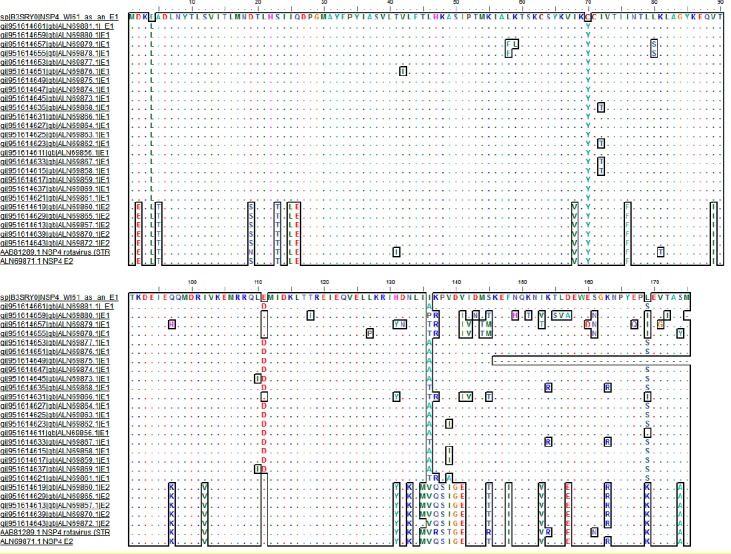
Multiple sequence alignments (MSAS) of protein NSP4 human rotavirus group A by accession number. For MSAS, muscle algorithm was applied, and E1 and E2 of *NSP4* genogroups were compared to WI61 and RV5 (Accession numbers: ABV53305.1 and AAB81289.1), respectively.

In the double-layered particle-binding site (161-175) of E1 and E2 genotypes, non-charged and charged aa (169 and 174) substitutions (S, K) were frequently detected, respectively (Fig. 1). In position 169 of E1 and E2 genotypes, amino acids serine and lysine were frequently detected. However, in some of the positions (162,164, 165, 166, 168, 170, and 173) of double-layered particle-binding region, conserved aa was detected. Diarrhea inducing motifs 114 to 135 in E1 and E2 genotypes in our samples had variations in some positions. Position 131 in NSP4 of non-avian RVs is histidine or tyrosine.

Viroporin domain of NSP4 sequence, which extends between 47 to 92 amino acids, contains two subdomains: pentalysine and amphipathic[8]. Pentalysine in our sequence contained lysine at positions 55, 59, 62, 66, and 69 as mentioned previously[8] (Fig. 1). Moreover, cysteine residues located at positions 63 and 71 of viroporin domain are conserved and play vital roles in disulfide bond

formation and NSP4 oligomerization[8]. In many protein sequences, sites of the E2 and E1 genotypes (2, 111, 157, and 163), replacement of acidic or basic aa has been noticed, but in some of the positions (26, 97, 133, 141, and 169), polar amino acid was substituted with non-polar aa (Fig. 1).

### NSP4, P, and G typing and gene constellation

**Fig. 2:**
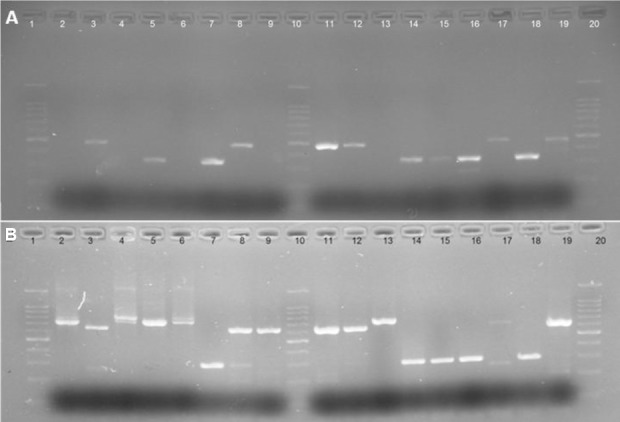
PCR pattern of G and P type human rotavirus. PCR amplicon of rotavirus typing (A) Representative agarose gel electrophoresis of PCR products of semi-multiplex P typing. Lanes 1, 10, and 20, 100 bp ladder; lane 18, RV4 (P8) representing positive control; lanes 3, 8, 11, 12, 17, and 19, P[4] genotype; lanes 5, 7, 14, 15, and 16, P[8] genotype; lane 2, negative control. Lanes 4, 9, and 13, negative results. (B) Representative agarose gel electrophoresis of PCR products of semi-multiplex G typing. Lanes 1, 10, and 20, 100 bp ladder; lane 2, RV4 (G1) representing positive control; lanes 4, 5, and 6, G1 genotype (749 bp); lanes 3, 8, 9, 11, 12, and 19, G2 genotype (652 bp); lanes 7, 14, 15, and 18, G9 (305 bp); lanes 17, 16, and 13, negative results.

Genotyping analysis of RV in 27 samples showed the following result: G1 in 7 cases (25.9%), G2 in 8 cases (29.7%), G3 in 2 cases (7.4%), and G9 in 10 cases (37%). The amplicon sizes of G1, G2, G3, G4, and G9 genotypes were 749, 652, 813, 584, and 305 bp, respectively (Fig. 2b). The G8 genotype was not detected in the samples, but mixed infections were found in three samples, two G1/G2 and one G2/G9. The emerging G9 genotype occurred in several countries with a frequency between 2.2 and 11.1%; however, it showed a high frequency in Turkey (40.5%), where it was the most common genotype[2].

The most frequent P types were P[8] in 17 (62.9%) samples, P[4] in 8 (29%), and P[6] in 1 (3%). Amplicon sizes of P[4], P[8], P[6], and P[9] genotypes were 484, 346, 260, and 392 bp, respectively; P[9] genotype was not identified (Fig. 2a). In all G/P samples, the constellation of G1/G9 and G2 was associated with P[8] and P[4], respectively. G3P [6] was identified in one of the samples, and in all samples, constellation of G1, 9/P[8]/E1 was shown. Besides, G2P[4] genotype was associated with E2 genotype but G3 with E1 genotype.

### Phylogenetic tree

**Fig. 3:**
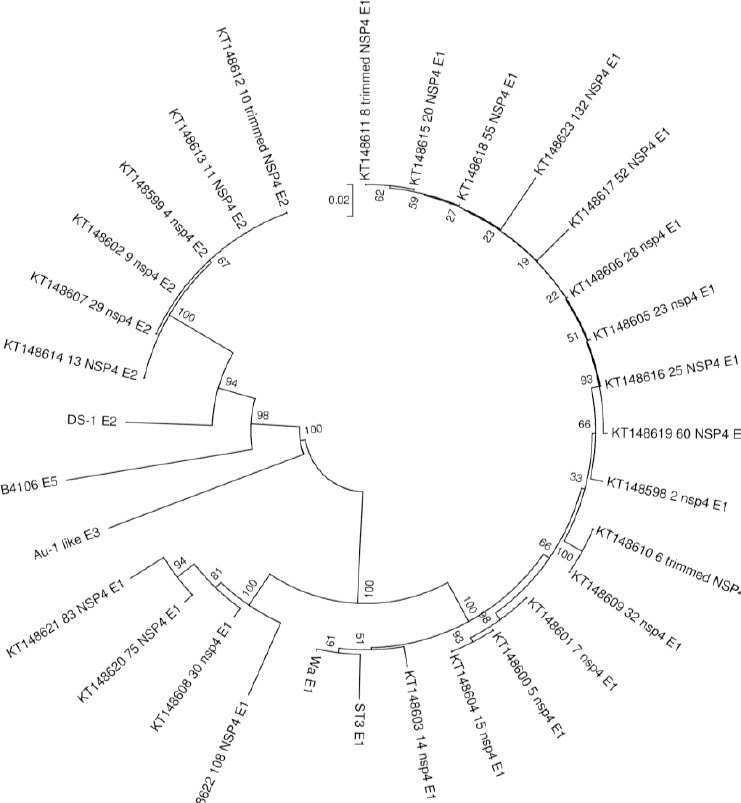
Phylogenetic tree of nucleotide sequences (nt 21-675, strain WA) of human RV strains genotyped and characterized in this study. Reference strains are named, and sample strains are indicated by their GenBank accession numbers. Bar show 0.02 substitutions per nucleotide. The most commonly detected RV genotypes in Iran were G4P[8] and G3P[6] genotypes alongside RV3 and McN13 prototype strains. The tree was inferred utilizing the nucleotide neighbor-joining algorithm with 200 bootstrapping replication of MEGA software.

Phylogenetic analysis confirmed the results obtained by sequencing analysis for *NSP4* genotyping assay (Fig. 3). Multiple sequence alignments were conducted by muscle algorithm using MEGA 6.0 software[22]. E1 and E2 were observed in separated clades and were also compared with some of the reference sequences in the GenBank.

### SDS-PAGE analysis of samples

**Fig. 4:**
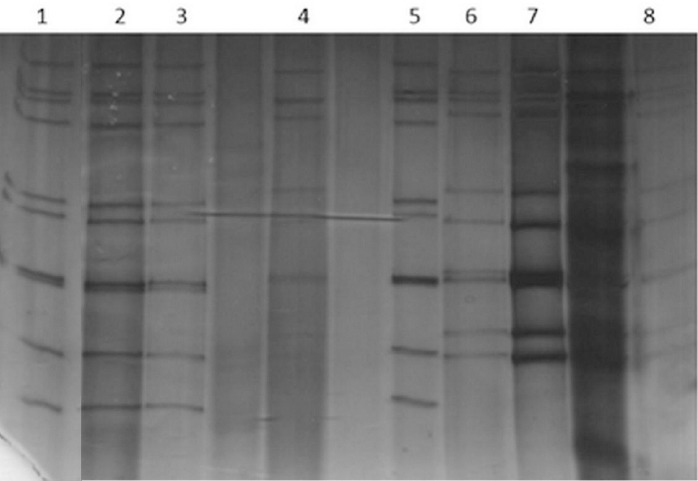
Electrophoretyping of RV from different genotypes. Lanes 1 and 5, G1P[8]; lanes 2 and 3, G1P[8]; lanes 4, 6, 7, and 8, G2P[4]. The short electrophoretic pattern in G2P[4] E2 genotype has been shown.

SDS-PAGE was carried out to represent the electrophoretic pattern of genotypes G and P and also to compare their patterns together. In the combination of G1/G9P[8], a similar pattern was detected (Fig. 4). Electrophoretic movement of VP4 and VP7 segments in these genotypes has been shown in the same position. The short electrophoretic pattern of G2/P4 genotype (samples 4, 6, 7, and 8) with a totally different pattern from G1/G9P[8] was presented. Moreover, in 10 segments of G2P[4] corresponding to E2 genotype a different pattern was presented.

## DISCUSSION

The aim of this study was the analysis of nucleotide and amino acid sequences of *NSP4* in RV-positive samples. In this report, the samples were genotyped based on G or P genotyping orresponding with VP7 and VP4 gene segments, and gene constellation of VP7, VP4, and *NSP4* was also compared.

Since *NSP4* is the most important and multi-functional protein of RV[6], analysis of this gene and its protein in clinical samples may be significant.

However, RV genome is segmented, and evolution of each segment can be analyzed for G or P typing[21,23]. As a result, genotyping of *NSP4* gene in the samples was carried out using RotaC2.0 web server, and E1 and E2 genotypes were identified. From 27 sequences samples, E1 (20 cases, ~77%) and E2 (6 cases, ~23%) were obtained. In general, E1 and E2 genotypes are dominant E genotypes in human RV samples[15,24]. Therefore, it could be assumed that the diversity of *NSP4* genes among human RV strains is restricted to two of the five *NSP4* genotypes described. This protein comprises multiple domains, and each domain has a specific function. Viroporin is one of the important domains of NSP4 enterotoxin protein, which comprises two motifs: pentalysine and amphipathic[8,25]. The presence of a conserved pentalysine domain among the NSP4 protein sequence has been demonstrated, and the alteration of this domain in clinical samples is probably associated with variation in the pathogenesis of virus.

Asparagine residues have been demonstrated to be glycosylated at positions 8 and 18[26]. In general, in N-linked glycosylation, Asparagine residues are located in a specific pattern sequence in the primary structure (Asn-X-Ser or Asn-X-Thr or in rare instances Asn-X-Cys). Among the E1 and E2 genotypes, the amino acid residue 8 in NSP4 protein was found in Asn-Tyr-Thr glycosylation pattern, while the amino acid number 18 was consistently observed in As-Asp-Thr pattern for E1 genotype and As-Ser-Thr pattern for E2 genotypes. Previously, experimental and sequence analysis has been suggested that all gene reassortants in RV are not random[27-30]; the existence of RV genogroups is the strongest evidence in favor of this argument. Moreover, protein-protein interactions in RV particles play an important role in the evolution of these viruses[27]. There is a strong association between P and G typing with E typing in RV samples.

Gene association G1, 9/P[8]/E1, and G2P[4]/E2 were detected, and no inter-genogroup reassorting containing VP7, VP4, and *NSP4* occurred in these conventional strains.

Based on previous epidemiological studies from Iran[31-42], there is a rare report on G9 genotype; however, in this study, G9 genotype (~37%) was detected in Khuzestan geographical region. Presently, G9 genotype has become the fifth most common genotype identified in humans[43]. In the samples used in our study, genotype G9 was associated with P[8], a genotype that is responsible for the majority of infections in the world. However, other VP4 genotypes such as P[4], P[6], P[11], and P[19] are also reported with G9. One of the major limitations of RV genotyping with WHO protocols is that G or P genotyping primers do not cover all samples with similar genotypes. Therefore, designing the conserved primers for non-typeable G and P genotypes are inevitable. In this study, we designed some primers to amplify the conserved region of VP4 and VP7 genes. Subsequently, the non-typeable RV samples were subjected to PCR amplification by those primers, and the PCR products were sequenced by Sanger sequencing method.
